# Anthropometry of Iranian Guidance School Students with Different Ethnicities: A Comparative Study

**DOI:** 10.1155/2015/893489

**Published:** 2015-10-08

**Authors:** Amir Houshang Mehrparvar, Rahmatollah Hafezi, Seyyed Jalil Mirmohammadi, Mehrdad Mostaghaci, Mohammad Hossein Davari

**Affiliations:** ^1^Occupational Medicine Department, Shahid Sadoughi University of Medical Sciences, Yazd 8175665563, Iran; ^2^Physical Medicine Department, Shahid Beheshti University of Medical Sciences, Tehran, Iran

## Abstract

*Objective*. We measured some anthropometric dimensions of Iranian guidance school students selected from different ethnicities. *Background*. Anthropometric dimensions are used for design of equipment, furniture, and clothing. Furniture with inappropriate design not fulfilling the users' anthropometric dimensions may have a negative effect on health. *Method*. A total of 7400 Iranian guidance school students aged 12–14 years entered the study and their static anthropometric dimensions were measured. Descriptive statistics such as mean, standard deviation, and key percentiles were calculated. All dimensions were compared among different ethnicities and different genders. *Results*. This study showed significant differences in a set of 22 anthropometric dimensions regarding gender, age, and ethnicity. *Conclusion*. According to the results of this study, difference between genders and among different ethnicities should be taken into account by designers and manufacturers of guidance school furniture. *Application*. This study has prepared a data bank of anthropometric dimensions of 12–14-year-old students which can be used as basic information to find appropriate dimensions of school furniture.

## 1. Introduction

In order to properly design different products, it is important to have access to anthropometric dimensions of the potential users [1–7].

Inappropriate dimensions of furniture and other products which do not match the users' body dimensions may lead to many adverse effects [3, 5, 8–12]. Students are among the populations at risk for musculoskeletal disorders as a result of incorrect posture, frequently caused by inappropriate school furniture [6, 11].

Appropriate design according to the target user groups and identification of the most important body dimensions are key issues in the design process [7]. There is a high prevalence of mismatch between anthropometric data and school furniture which is a factor implicated in causing low back pain [13].

Several researches have worked on anthropometric dimensions of people in different ages in many countries with different races or ethnicities [9, 14–20].

It is documented that such factors as age, gender, race, ethnicity, nutrition, and geographic area will affect anthropometric dimensions [4, 21–24].

Race refers to grouping of people according to biological characteristics, while ethnicity also encompasses additional cultural factors [25]. Some races consist of different ethnicities with probably different cultural, geographical, economic, and nutritional properties which may eventually create different anthropometric dimensions [5, 24, 26–29]. Anthropometric dimensions in a population may change continuously [14]. Smith and Norris have shown a significant change in body size of the UK children during the past thirty years [30].

There are six ethnicities in different parts of Iran (i.e., Arab, Baluch, Fars, Kurd, Lor, and Turk) with different cultural, economic, nutritional, and geographical characteristics. Turk and Kurd population live in a naturally rich area with cold and damp weather in west and northwest of Iran, and Baluch population live in a deprived area with a hot and dry weather in southeast of Iran. It is said that Kurd and Turk population are naturally larger in body size than other ethnicities. The authors have found these differences in their previous study on primary school children in different Iranian ethnicities [24].

Anthropometric studies on Iranian population are few and most of them with small sample size [31, 32]. To the best of our knowledge the only large studies for measurement of anthropometric dimensions of students were our previous studies on Iranian primary school children and university students [24, 33].

### 1.1. Purpose

Lack of national anthropometric data leads to the design of clothing, shoes, and furniture based upon anthropometric dimensions of other populations, which may not represent the body sizes of the real population. This study was designed to measure some static anthropometric dimensions in Iranian guidance school children considering ethnic differences.

## 2. Material and Methods

This was a cross-sectional study conducted on 7400 guidance school students aged 12–14 years from different ethnicities in Iran. The students were assigned in each age category according to the information of their identity card; for example, a student was considered to be 12 years old when he (she) was born in the year 1377* Anno Persico* (between 21.3.1998 and 20.3.1999 AD).

**Figure 1:**
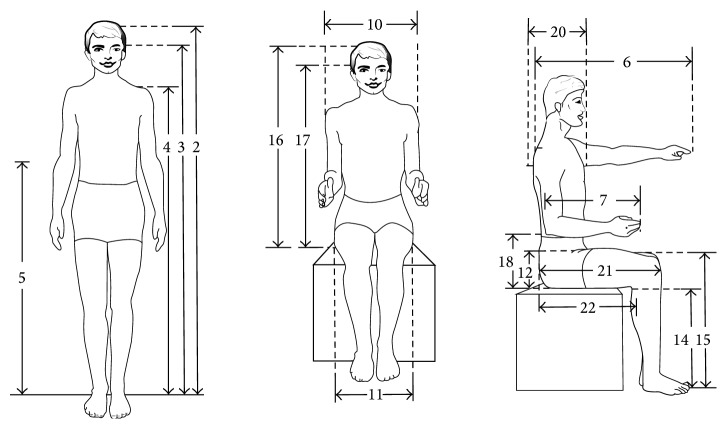


The dimensions which were measured included the following: weight, standing vertical dimensions (height, eye height, shoulder height, and elbow height), sitting vertical dimensions (popliteal height, knee height, sitting height, eye height, and elbow height), horizontal dimensions (arm length, forearm length, forearm-forearm distance, elbow-elbow distance, shoulder width, buttock width, buttock-knee length, and buttock-popliteal length), depths (chest and abdomen), and thicknesses (one-thigh and two-thigh) [24].  Figure 1 shows the measured dimensions.

**Figure 2:**
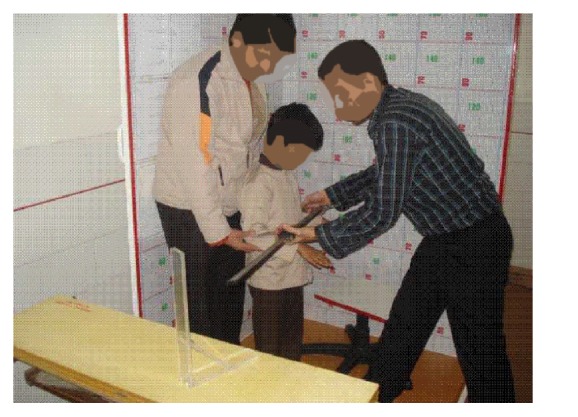


The subjects stood and sat in standard postures for measurement of standing and sitting dimensions (27, 28). Six groups of technicians who were trained for measurements in a planned course performed the measurements using similar techniques. The groups consisted of an observer and two recorders. The dimensions were repeated for 7% of subjects by two other observers blinded to the previous measurements. Subjects entered the study wearing home clothing without shoes (Figure 2).

### 2.1. Subjects

The study sample included 7400 subjects (3560 boys and 3840 girls) from six ethnicities (1271 Fars, 1234 Kurd, 1342 Lor, 1192 Baluch, 1200 Turk, and 1191 Arab students). The details of the number of subjects are presented in Table 1. Measurements were made during a 4-month period in 2010.

We obtained an informed written consent from parents and oral consent from students after explanation of the procedure.

### 2.2. Analysis

Descriptive statistics (mean, standard deviation, and 5, 50, and 95 percentiles) were measured for each dimension regarding gender, age, and ethnicity. The dimensions were compared between two genders and among six ethnicities in each age group. Student's *t*-test and one-way ANOVA were used for the comparison of means between two genders and among six ethnicities, respectively.

## 3. Results

In this study, 7400 subjects (3560 boys and 3840 girls) aged 12 to 14 years in six ethnicities were assessed.  Table 2 shows means of 22 anthropometric dimensions of guidance school children in 6 different Iranian ethnicities.

Table 3 shows key percentiles (i.e., 5, 50, and 95) for 6 most important anthropometric dimensions (i.e., weight, body height, standing eye height, standing shoulder height, standing elbow height, and buttock-popliteal length) in different ethnicities.

There was a significant difference between males and females in most dimensions.  Table 4 shows the key percentiles of six more important anthropometric dimensions and Table 5 shows the comparison of dimensions regarding gender.

There was a significant difference between ethnicities in all anthropometric dimensions. *p* values for difference among ethnicities were less than 0.001 for all dimensions except for height in 12-year-old girls (*p* = 0.260), sitting height in 12-year-old girls (*p* = 0.519), and sitting eye height in 13-year-old girls (*p* = 0.030).

## 4. Discussion

Today, it is important to measure anthropometric dimensions as a key step for design process. Such variables as age, gender, and ethnicity affect these dimensions, so it is critical to consider these variables for preparation of anthropometric databases.

In this study we measured the anthropometric dimensions of students aged 12 to 14 years from different ethnicities in Iran. The results showed a significant difference between two genders in all age groups and among all ethnicities for most of the dimensions.

In this study, 12- and 13-year-old girls' weight was significantly more than boys which was opposite in 14-year-old subjects. In 12-year-old subjects, girls had larger heights and upper extremity lengths, although boys showed larger lower extremity heights and lengths. In 13- and 14-year-old subjects, most anthropometric dimensions were significantly larger in boys except for depths and dimensions related to buttocks which is explainable by the size of breasts and buttocks in girls due to puberty.

In this study, Turk boys had the largest dimensions in most of the measured dimensions except for abdominal depth, upper extremity distances and widths, lower extremity, and sitting heights which were larger in Fars ethnicity, although in some of them such as forearm-forearm distance the difference was negligible. Most dimensions were smaller in Baluch boys than other ethnicities except for most upper extremity dimensions and some lower extremity heights.

In about one-third of dimensions Arab girls had the largest measures and in other dimensions Kurds and Turks showed larger dimensions.

There is a considerable difference among different Iranian ethnicities regarding genetic characteristics, climate, geographical area, and socioeconomic characteristics which may affect anthropometric dimensions. For example, Turks and Kurds live in a naturally rich area, but Baluchis live in a province which is naturally deprived, so some differences are probably due to these issues.

There are some studies in different populations for measurement of anthropometric dimensions. Mokdad and Al-Ansari measured 44 anthropometric dimensions of Bahraini children aged 6–12 years. They found significant difference between two genders in many dimensions [34]. A total of 50 anthropometric dimensions were measured among Mexican children and a significant difference among different populations was found [16].

The difference between races or ethnicities regarding anthropometric dimensions has also been identified in some studies. Rosnah et al. found a significant difference between Malays and non-Malays which is in agreement with the results of current study [35]. Jahanshahi et al. found that ethnicity affects facial anthropometric dimensions in an Iranian population [27]. Lin et al. also found a significant difference among four East Asian populations [5].

Although climatic, nutritional, and economic factors are significantly different in different populations and countries, one of the important factors contributing to anthropometric differences is race or ethnicity.

Anthropometric dimensions of the study children were different from other populations. Anthropometric dimensions of Greek, American, and Mexican children were more than Iranian children [16, 17, 19] and these dimensions in Vietnamese children were less than Iranian children [9].

The anthropometric dimensions we measured in this study can be used to design school furniture matched to our population. It is recommended that the number of anthropometric dimensions be increased to create databases used to design clothing, shoes, and other products. It is also recommended that these measurements be repeated to seek for temporal trends.

This study had some limitations. We tried to select real native students in each ethnicity, but some hybrid students may have entered the study and we might not have detected these subjects.

## 5. Conclusions

In this study we found significantly different anthropometric dimensions in different Iranian ethnicities. This makes it necessary to pay attention to these differences when school furniture is designed. The results of this study showed that students need furniture specifically designed and manufactured considering their ethnicity, gender, and age. It is obvious that designing school furniture separately for each ethnicity is very difficult, so a practical approach is to design adjustable furniture using the anthropometric dimension measured in this study.

## Key Points

- Anthropometric dimensions of the users should be put in mind when determining the dimensions of school furniture.
- This study showed a significant gender difference in all anthropometric dimensions.
- This study showed a significant difference among different ethnicities in most anthropometric dimensions.

## Figures and Tables

**Table 1:** Definition of anthropometric data.

Anthropometric dimensions	Definition
(1) Weight	Body weight
(2) Body height	Vertical distance from the floor to the vertex (i.e., the crown of the head)
(3) Eye height (standing)	Vertical distance from the standing surface to the inner canthus of the eye
(4) Shoulder height (standing)	Vertical distance from the standing surface to the shoulder
(5) Elbow height (standing)	Vertical distance from the standing surface to the underside of the elbow
(6) Arm length	Difference between shoulder height and elbow height
(7) Forearm length	Distance between acromion and tip of the middle finger
(8) Forearm-forearm distance	Maximum distance between two forearms
(9) Elbow-elbow distance	Distance between two acromions in standard sitting position
(10) Shoulder width	Maximum shoulder width in standing position
(11) Buttock width	Maximum buttock width in sitting position
(12) One-thigh thickness	Maximum thickness of the thigh
(13) Two-thigh thickness	Maximum two-thigh thickness when right thigh rests over left thigh
(14) Popliteal height (sitting)	Vertical distance from the floor to the popliteal angle at the underside of the knee where the tendon of the biceps femoris muscle is inserted into the lower leg
(15) Knee height (sitting)	Vertical distance from the floor to the upper surface of the knee in sitting position
(16) Sitting height	Vertical distance from the sitting surface to the vertex
(17) Eye height (sitting)	Vertical distance from the sitting surface to the inner canthus of the eye
(18) Elbow height (sitting)	Vertical distance from the seat surface to the underside of the elbow
(19) Abdominal depth	Maximum horizontal distance from the vertical reference surface to abdominal front in sitting position
(20) Chest depth	Maximum horizontal distance from the vertical reference plane to the front of the chest in men or breast in women
(21) Buttock-knee length	Horizontal distance from the back of the uncompressed buttocks to the front of the kneecap
(22) Buttock-popliteal length	Horizontal distance from the back uncompressed buttocks to the popliteal angle, at the back of the knee, where the back of the lower legs meets the underside of the thigh

**Table 2:** Sample size of the subjects in different ethnicities and age groups.

	Age (year)						Total		
Ethnicity	12		13		14				
	Boys	Girls	Boys	Girls	Boys	Girls	Boys	Girls	Total
Fars	225	166	223	201	230	246	708	613	1301
Kurd	152	176	227	204	199	226	578	606	1184
Lor	227	268	227	236	184	200	638	704	1342
Baluch	126	233	150	252	223	208	499	737	1192
Turk	208	186	203	211	184	208	595	605	1200
Arab	206	132	237	212	129	275	572	619	1191
Total	1144	1161	1277	1316	1149	1363	3560	3840	7400

**Table 3:** Mean anthropometric dimensions in Iranian ethnicities.

Dimensions	Age	Ethnicity											
		Fars		Kurd		Lor		Baluch		Turk		Arab	
		Boys	Girls	Boys	Girls	Boys	Girls	Boys	Girls	Boys	Girls	Boys	Girls
Weight (kg)	12	41.82	43.10	37.49	42.51	39.08	41.62	*32.13*	*37.83*	**44.26**	42.12	40.67	**44.89**
	13	47.65	48.11	43.79	48.18	44.37	47.61	*37.39*	*40.74*	**49.36**	46.99	47.45	**48.58**
	14	54.66	**52.22**	49.74	50.65	52.58	51.54	*41.50*	*44.05*	**57.80**	49.98	51.81	52.13

Body height (mm)	12	1490.86	1497.77	1456.08	1499.94	1458.01	1490.85	*1425.42*	*1484.14*	**1512.40**	1501.73	1492.57	**1507.19**
	13	1559.82	1534.32	1524.44	**1543.77**	1536.12	1541.77	*1505.20*	*1516.03*	**1591.87**	1536.91	1563.90	1537.40
	14	1644.30	1570.52	1596.20	1572.03	1610.86	1572.75	*1546.52*	*1540.60*	**1659.72**	**1573.89**	1608.75	1559.33

Standing eye height (mm)	12	1375.04	1376.95	1335.42	1380.85	1342.33	1372.79	*1315.47*	*1372.70*	**1393.55**	1382.12	1359.73	**1383.48**
	13	1444.75	1415.34	1402.11	**1422.23**	1422.51	1418.64	*1392.93*	*1402.20*	**1471.36**	1416.91	1435.21	1417.21
	14	1533.58	**1453.80**	1472.63	1449.84	1499.89	1452.45	*1435.80*	*1431.15*	**1543.58**	1451.46	1490.81	1435.51

Standing shoulder height (mm)	12	1226.35	1242.77	1184.40	1230.11	1198.76	1224.29	*1169.08*	*1217.87*	**1244.18**	1222.74	1216.82	**1246.66**
	13	1286.54	**1276.84**	1249.53	1271.05	1267.62	1272.54	*1241.46*	*1243.80*	**1311.57**	1242.91	1280.25	1272.66
	14	1361.47	**1310.18**	1313.06	1293.96	1338.04	1300.85	*1275.40*	*1269.59*	**1376.11**	1277.86	1328.06	1292.00

Standing elbow height (mm)	12	911.77	922.54	881.08	921.85	913.61	*903.89*	*880.41*	**947.74**	**936.98**	918.15	932.59	925.42
	13	951.90	946.31	*929.16*	949.55	961.54	*934.32*	929.19	**973.69**	**987.82**	934.33	982.45	955.97
	14	1016.00	968.78	975.64	958.71	1010.05	*957.90*	*961.47*	**986.44**	**1035.43**	962.66	1005.96	970.58

Chest depth (mm)	12	175.79	182.57	174.40	197.53	163.52	178.91	*161.12*	*175.33*	**186.11**	200.01	173.30	**201.15**
	13	185.68	197.94	183.12	212.41	172.73	192.97	*172.33*	*188.36*	**193.55**	**214.96**	187.98	208.59
	14	191.57	206.81	192.82	**220.14**	184.07	*197.00*	*174.03*	197.62	**203.95**	224.55	197.87	215.22

Abdominal depth (mm)	12	**170.65**	164.63	163.26	175.73	153.65	**178.87**	*144.39*	*157.27*	165.12	164.70	162.71	171.10
	13	**178.15**	169.24	173.63	185.73	159.82	**188.78**	*150.42*	*158.81*	169.62	169.97	173.89	175.76
	14	**184.40**	170.61	179.17	186.06	170.43	**189.80**	*155.45*	*161.37*	179.63	172.75	172.24	175.67

Arm length (mm)	12	298.61	316.74	304.09	313.38	*294.05*	307.95	298.36	311.76	**312.76**	*306.25*	294.38	**318.20**
	13	313.87	**325.83**	322.29	324.85	*312.64*	316.47	316.03	318.21	**330.14**	*314.11*	317.24	324.43
	14	330.11	**333.23**	339.14	329.54	329.07	322.39	327.34	326.64	**348.40**	*319.85*	*323.58*	329.68

Forearm length (mm)	12	387.78	*384.21*	389.38	400.02	380.88	394.36	*375.95*	385.21	**405.22**	396.41	388.52	**402.93**
	13	413.76	397.42	410.39	409.96	*404.97*	409.35	406.22	*391.71*	**428.85**	409.48	417.16	**413.42**
	14	437.87	*402.45*	432.13	**417.43**	426.03	412.39	*419.62*	405.94	**448.92**	415.45	426.31	416.62

Forearm-forearm distance (mm)	12	**398.77**	376.28	357.38	348.48	348.94	*325.97*	*320.41*	339.58	395.67	**402.30**	336.00	372.69
	13	**418.32**	388.50	376.15	359.24	356.16	343.11	*337.99*	*343.08*	405.85	**418.39**	357.01	389.10
	14	**439.14**	396.52	396.49	363.65	381.08	*335.16*	*347.75*	356.49	436.97	**420.52**	364.43	398.10

Elbow-elbow distance (mm)	12	369.51	*315.78*	**370.35**	361.05	330.13	349.27	315.69	336.93	359.99	358.58	*298.33*	**373.81**
	13	385.50	*328.14*	**392.87**	373.74	343.52	370.31	330.41	341.54	372.11	373.66	*323.21*	**388.05**
	14	403.61	*334.42*	**413.43**	380.86	363.91	385.85	*342.07*	357.91	395.26	377.19	348.88	**395.76**

Shoulder width (mm)	12	**349.75**	**358.21**	335.59	338.26	*321.23*	335.04	321.56	339.74	345.89	*318.35*	328.16	351.73
	13	**374.75**	**371.54**	351.41	352.25	338.28	353.13	*337.21*	348.54	364.68	*329.78*	353.24	362.76
	14	**391.89**	**381.17**	367.98	357.77	363.04	355.35	*350.30*	362.66	387.34	*338.25*	360.29	373.77

Buttock width (mm)	12	271.13	287.44	288.48	306.60	272.24	281.33	*257.47*	*270.18*	**293.11**	**311.90**	270.42	303.22
	13	285.79	298.86	302.34	326.50	286.74	299.75	*274.60*	*279.53*	**307.81**	**327.75**	291.82	320.23
	14	298.46	311.58	317.28	331.76	310.21	300.17	*283.53*	*289.00*	**328.26**	**340.32**	293.12	331.32

One-thigh thickness (mm)	12	**112.87**	98.82	105.74	**118.14**	99.86	*78.13*	*93.63*	116.91	108.76	111.33	97.82	112.74
	13	**121.78**	105.33	111.60	117.27	107.86	*90.30*	*94.31*	113.33	115.70	117.60	107.52	**122.76**
	14	**126.73**	107.85	117.96	**127.38**	120.54	*84.99*	*100.47*	117.38	124.25	122.42	110.52	123.89

Two-thigh thickness (mm)	12	**235.50**	205.66	197.12	**225.26**	*187.18*	188.58	*181.69*	198.63	220.99	199.89	216.83	195.36
	13	**237.34**	215.24	207.51	**240.77**	196.96	204.00	*181.67*	*196.53*	226.23	209.41	240.14	203.31
	14	**246.63**	221.75	218.27	**243.07**	216.95	202.79	*187.47*	*202.75*	245.96	216.77	234.86	208.19

Popliteal height (mm)	12	*352.09*	356.37	372.23	361.59	375.24	*355.82*	**382.83**	364.77	381.76	**370.56**	372.96	370.15
	13	*362.44*	*361.22*	391.80	365.01	400.04	368.60	399.86	367.46	**400.74**	373.84	387.65	**381.98**
	14	*380.76*	*366.19*	410.10	374.44	**415.21**	368.53	410.14	380.50	413.07	377.69	403.25	**384.58**

Knee height (mm)	12	*451.95*	459.00	461.38	454.28	452.37	*441.41*	471.86	**467.86**	**481.35**	452.87	479.10	437.53
	13	*471.68*	467.32	486.19	458.46	480.35	458.55	480.65	**473.10**	502.57	458.00	**504.30**	*449.05*
	14	493.72	475.90	509.29	468.12	503.09	466.16	*493.60*	**483.86**	**525.35**	467.28	513.52	*457.85*

Buttock-popliteal length (mm)	12	*364.23*	408.00	**406.28**	**416.28**	370.61	398.52	364.81	*389.31*	387.45	396.56	399.81	405.98
	13	*382.48*	414.51	**427.20**	**428.27**	392.33	414.55	388.41	*404.90*	406.16	408.33	424.70	418.36
	14	407.76	425.40	**446.78**	**437.97**	415.54	422.28	*403.49*	*409.54*	426.31	421.20	431.78	429.32

Buttock-knee length (mm)	12	486.69	505.09	482.40	508.31	466.82	**508.94**	*452.87*	*472.63*	501.40	489.27	**501.77**	506.70
	13	508.08	518.57	506.97	520.51	493.52	527.25	*478.76*	*490.34*	527.35	503.16	**532.63**	**523.74**
	14	533.86	533.07	529.34	531.68	523.58	**535.22**	*493.40*	*500.15*	**552.88**	516.65	537.82	531.57

Sitting height (mm)	12	764.40	785.48	769.60	777.89	**771.63**	766.08	*711.96*	*757.13*	769.91	787.48	750.34	**797.83**
	13	801.90	807.71	799.11	812.21	799.69	791.35	*751.96*	*778.63*	**815.73**	809.08	779.43	**819.52**
	14	**846.56**	**833.61**	828.51	824.04	838.42	806.45	*780.54*	*793.99*	843.50	833.32	807.22	829.08

Sitting eye height (mm)	12	649.02	666.29	652.16	665.79	**656.78**	654.02	*611.73*	*643.48*	650.04	**674.43**	631.37	673.75
	13	690.35	691.79	679.58	697.27	686.91	671.35	*649.86*	*666.78*	**697.43**	695.54	657.72	**698.72**
	14	738.26	716.01	709.34	705.27	725.32	*678.15*	*674.86*	681.71	**726.78**	**719.01**	688.72	708.42

Sitting elbow height (mm)	12	*173.00*	199.24	195.98	189.32	**203.30**	*174.70*	179.40	**217.07**	201.40	205.59	180.52	204.00
	13	*186.09*	208.08	204.85	196.01	212.59	*182.92*	190.27	**230.83**	**232.80**	214.28	180.71	212.76
	14	200.36	222.90	211.33	200.24	221.68	*185.70*	205.21	**238.60**	**231.57**	231.65	*186.74*	220.41

Bold and italic prints show the highest and lowest values, respectively, in each age group and gender.

**Table 4:** Key percentiles of six anthropometric dimensions.

Dimension	Ethnicity	Sex	Age								
			12			13			14		
			Percentile			Percentile			Percentile		
			5	50	95	5	50	95	5	50	95
Weight (Kg)	Fars	Boys	28.16	39.10	61.94	31.00	45.50	68.90	36.90	52.85	80.63
		Girls	27.71	42.55	63.91	32.11	46.70	69.80	38.43	50.40	71.00
	Lor	Boys	28.00	37.00	56.20	30.00	42.00	65.60	35.00	49.50	77.75
		Girls	28.00	41.00	59.55	31.85	46.00	67.15	37.05	50.00	68.90
	Kurd	Boys	28.19	35.65	54.51	30.74	41.80	64.05	34.20	48.10	73.10
		Girls	28.58	41.40	62.36	33.00	47.05	68.80	38.73	49.00	67.29
	Turk	Boys	31.09	41.80	66.05	34.42	47.70	70.00	41.17	55.65	85.45
		Girls	29.63	40.75	58.93	32.00	46.40	65.96	36.10	48.40	68.77
	Baluch	Boys	25.00	31.00	44.30	27.05	36.00	53.00	28.04	40.00	60.80
		Girls	26.70	36.0	52.00	28.00	40.00	56.00	31.45	43.00	59.00
	Arab	Boys	28.00	38.75	58.00	32.45	46.00	68.00	35.00	51.00	76.00
		Girls	29.79	43.50	63.74	33.63	46.85	75.17	39.36	50.80	69.86

Body height (mm)	Fars	Boys	1370.00	1490.00	1620.00	1410.00	1560.00	1710.00	1495.50	1645.00	1784.50
		Girls	1380.25	1510.00	1595.00	1430.00	1540.00	1634.50	1481.75	1570.00	1655.00
	Lor	Boys	1360.00	1460.00	1560.00	1400.00	1540.00	1680.00	1462.50	1600.00	1750.00
		Girls	1360.00	1495.00	1610.00	1420.00	1550.00	1641.50	1480.00	1570.00	1679.50
	Kurd	Boys	1360.00	1445.00	1606.75	1395.00	1525.00	1683.00	1445.00	1595.00	1740.00
		Girls	1390.00	1500.00	1620.75	1437.50	1540.00	1655.00	1486.75	1570.00	1655.00
	Turk	Boys	1382.25	1510.00	1662.75	1456.00	1590.00	1734.00	1530.00	1665.00	1780.00
		Girls	1370.00	1505.00	1613.25	1423.00	1540.00	1640.00	1482.25	1575.00	1672.75
	Baluch	Boys	1300.00	1420.00	1569.50	1360.00	1502.50	1687.25	1390.00	1550.00	1700.00
		Girls	1367.00	1490.00	1610.00	1400.00	1520.00	1640.00	1442.25	1550.00	1630.00
	Arab	Boys	1370.00	1490.00	1636.50	1420.00	1560.00	1715.00	1442.50	1620.00	1740.00
		Girls	1374.75	1510.00	1601.75	1425.00	1540.00	1633.50	1450.00	1560.00	1660.00

Standing eye height (mm)	Fars	Boys	1255.00	1370.00	1498.50	1295.00	1450.00	1600.00	1390.00	1530.00	1684.50
		Girls	1248.50	1385.00	1475.00	1310.00	1420.00	1505.00	1360.00	1450.00	1550.00
	Lor	Boys	1240.00	1340.00	1450.00	1280.00	1430.00	1570.00	1352.50	1495.00	1640.00
		Girls	1240.00	1370.00	1490.00	1300.00	1420.00	1521.50	1370.5	1450.00	1560.00
	Kurd	Boys	1240.00	1330.00	1473.50	1272.00	1400.00	1552.00	1335.00	1475.00	1615.00
		Girls	1264.25	1375.00	1500.00	1316.25	1420.00	1542.50	1353.50	1450.00	1540.00
	Turk	Boys	1255.00	1390.00	1538.25	1330.00	1480.00	1623.00	1411.25	1550.00	1673.75
		Girls	1253.50	1390.00	1485.00	1310.00	1425.00	1520.00	1352.25	1450.00	1555.00
	Baluch	Boys	1186.75	1310.00	1466.00	1245.50	1382.50	1574.50	1270.00	1440.00	1600.00
		Girls	1250.00	1370.00	1513.00	1266.50	1405.00	1540.00	1332.25	1437.50	1525.50
	Arab	Boys	1230.00	1350.00	1530.00	1289.00	1430.00	1600.00	1305.00	1505.00	1635.00
		Girls	1240.00	1390.00	1481.75	1300.00	1420.00	1520.00	1325.00	1435.00	1535.00

Standing shoulder height (mm)	Fars	Boys	1110.00	1220.00	1345.00	1152.00	1290.00	1414.00	1235.50	1360.00	1480.00
		Girls	1125.00	1250.00	1340.00	1170.50	1285.00	1365.00	1231.75	1310.00	1395.00
	Lor	Boys	1104.00	1200.00	1296.00	1120.00	1270.00	1400.00	1200.00	1340.00	1467.50
		Girls	1094.50	1230.00	1330.00	1170.00	1280.00	1370.00	1220.00	1300.00	1399.50
	Kurd	Boys	1095.00	1175.00	1310.50	1132.00	1250.00	1394.00	1180.00	1310.00	1455.00
		Girls	1119.25	1230.00	1340.75	1171.25	1260.00	1377.50	1213.50	1295.00	1383.25
	Turk	Boys	1135.00	1245.00	1380.50	1190.00	1310.00	1440.00	1240.00	1380.00	1495.00
		Girls	1106.75	1222.50	1323.25	1140.00	1240.00	1340.00	1190.00	1275.00	1380.00
	Baluch	Boys	1050.00	1170.00	1306.50	1105.50	1240.00	1410.00	1120.00	1290.00	1428.00
		Girls	1110.00	1220.00	1336.50	1126.50	1250.00	1353.50	1184.50	1270.00	1350.00
	Arab	Boys	1110.00	1210.00	1340.00	1150.00	1280.00	1420.00	1180.00	1340.00	1440.00
		Girls	1123.00	1250.00	1333.50	1175.00	1272.50	1360.00	1199.00	1300.00	1380.00

Standing elbow height (mm)	Fars	Boys	820.00	910.00	1000.00	840.00	950.00	1040.00	911.00	1020.00	1110.00
		Girls	836.50	925.00	1005.00	870.25	950.00	1020.00	900.00	970.00	1035.00
	Lor	Boys	830.00	910.00	1000.00	860.00	960.00	1080.00	910.00	1010.00	1100.00
		Girls	804.50	900.00	990.00	840.00	940.00	1020.00	900.00	950.00	1030.00
	Kurd	Boys	808.25	875.00	973.50	840.00	925.00	1025.00	880.00	980.00	1070.00
		Girls	840.00	920.00	1006.00	880.00	945.00	1034.00	900.00	955.00	1020.00
	Turk	Boys	840.00	935.00	1040.00	901.00	990.00	1088.00	877.50	1020.00	1100.00
		Girls	830.00	920.00	1000.00	843.00	940.00	1012.00	902.25	960.00	1040.00
	Baluch	Boys	783.50	880.00	976.50	832.75	930.00	1054.50	840.00	970.00	1088.0
		Girls	867.00	950.00	1050.00	896.50	980.00	1070.00	904.50	990.00	1080.00
	Arab	Boys	820.00	925.00	1056.50	880.00	980.00	1090.00	920.00	1040.00	1130.00
		Girls	780.00	940.00	1000.00	890.00	955.00	1031.75	900.00	970.00	1050.00

Buttock-popliteal length (mm)	Fars	Boys	320.00	362.00	418.00	330.20	380.00	434.00	350.55	408.00	464.90
		Girls	355.40	411.00	458.30	365.40	415.00	465.00	379.35	424.00	471.00
	Lor	Boys	330.00	370.00	420.00	344.00	390.00	450.00	370.00	410.00	477.50
		Girls	443.00	512.50	568.55	358.80	412.00	476.60	375.00	420.00	472.85
	Kurd	Boys	369.00	404.50	449.70	373.80	428.00	480.60	396.00	446.00	499.00
		Girls	357.80	418.50	474.90	369.75	427.50	484.00	380.00	438.00	488.00
	Turk	Boys	342.00	387.00	441.20	350.20	408.00	450.00	383.00	425.50	473.25
		Girls	359.00	394.50	432.00	369.10	409.50	452.00	386.00	422.00	458.00
	Baluch	Boys	319.70	363.50	421.25	327.75	387.00	448.45	348.00	402.50	473.00
		Girls	326.20	392.00	437.90	343.90	407.00	459.00	353.00	410.00	460.55
	Arab	Boys	326.75	400.00	470.00	369.50	420.00	490.00	362.50	430.00	490.00
		Girls	338.38	406.00	456.70	363.24	418.55	468.36	387.26	428.50	476.84

**Table 5:** Comparison of anthropometric dimensions between two genders.

Dimensions	Age (year)								
	12			13			14		
	Boys	Girls	*p*	Boys	Girls	*p*	Boys	Girls	*p*
Weight (kg)	39.87	41.66	<0.001	45.39	46.52	0.012	51.10	50.25	0.072
Body height (mm)	1476.74	1495.47	<0.001	1548.67	1534.53	<0.001	1610.12	1564.79	<0.001
Standing eye height (mm)	1357.33	1377.30	<0.001	1429.47	1415.04	<0.001	1595.45	1445.44	<0.001
Standing shoulder height (mm)	1210.52	1228.82	<0.001	1274.02	1262.73	<0.001	1331.22	1291.32	<0.001
Standing elbow height (mm)	912.94	922.81	<0.001	958.31	949.54	<0.001	999.61	967.63	<0.001
Chest depth (mm)	172.98	187.45	<0.001	183.02	201.90	<0.001	188.74	210.58	<0.001
Abdominal depth (mm)	160.97	168.87	<0.001	168.62	174.09	<0.001	173.51	175.92	0.058
Arm length (mm)	300.22	311.69	<0.001	318.65	320.43	0.071	333.17	327.26	<0.001
Forearm length (mm)	388.62	393.23	<0.001	413.74	404.92	<0.001	431.91	411.77	<0.001
Forearm-forearm distance (mm)	362.91	356.85	0.003	376.65	372.02	0.017	395.98	379.94	<0.001
Elbow-elbow distance (mm)	341.33	348.07	<0.001	358.98	362.27	0.090	375.04	369.22	0.004
Shoulder width (mm)	334.52	339.01	0.001	353.95	352.74	0.364	370.78	362.64	<0.001
Buttock width (mm)	276.07	291.18	<0.001	292.26	307.68	<0.001	304.88	318.17	<0.001
One-thigh thickness (mm)	103.77	105.71	0.064	110.57	113.89	0.001	116.91	116.28	0.521
Two-thigh thickness (mm)	208.88	201.19	<0.001	216.91	210.75	<0.001	224.05	216.12	<0.001
Popliteal height (mm)	371.82	362.56	<0.001	389.71	369.69	<0.001	404.71	375.55	<0.001
Knee height (mm)	465.70	452.58	<0.001	487.95	461.04	<0.001	505.18	467.97	<0.001
Buttock-popliteal length (mm)	381.77	401.25	<0.001	404.65	414.44	<0.001	420.62	424.76	0.002
Buttock-knee length (mm)	483.83	497.61	<0.001	509.46	513.36	0.017	527.07	525.32	0.242
Sitting height (mm)	759.22	775.88	<0.001	793.01	802.02	<0.001	824.39	821.03	0.087
Sitting eye height (mm)	643.93	660.97	<0.001	677.98	685.96	<0.001	711.48	702.42	<0.001
Sitting elbow height (mm)	189.27	197.21	<0.001	201.15	207.82	<0.001	210.07	216.91	<0.001
